# Exosome-transmitted long non-coding RNA *SENP3-EIF4A1* suppresses the progression of hepatocellular carcinoma

**DOI:** 10.18632/aging.103302

**Published:** 2020-06-27

**Authors:** Jianchu Wang, Jian Pu, Ying Zhang, Tianwei Yao, Zongjiang Luo, Wenchuan Li, Guidan Xu, Juan Liu, Wujun Wei, Yibin Deng

**Affiliations:** 1 Department of Hepatobiliary Surgery, Affiliated Hospital of Youjiang Medical University for Nationalities, Guangxi Zhuang Autonomous Region, Baise 533000, China; 2 Clinic Medicine Research Center of Hepatobiliary Diseases, Affiliated Hospital of Youjiang Medical University for Nationalities, Guangxi Zhuang Autonomous Region, Baise 533000, China; 3 Library of Youjiang Medical University for Nationalities, Guangxi Zhuang Autonomous Region, Baise 533000, China; 4 Department of Infectious Diseases, Affiliated Hospital of Youjiang Medical University for Nationalities, Guangxi Zhuang Autonomous Region, Baise 533000, China; 5 Centre for Medical Laboratory Science, Affiliated Hospital of Youjiang Medical University for Nationalities, Guangxi Zhuang Autonomous Region, Baise 533000, China

**Keywords:** hepatocellular carcinoma, SENP3-EIF4A1, exosomes, biomarker, ceRNA

## Abstract

Extracellular communication mediated by exosomes in a tumor microenvironment can substantially affect tumor progression. However, the effect of exosomal long non-coding RNA *SENP3-EIF4A1* on hepatocellular carcinoma (HCC) is still unclear. In this study, *SENP3-EIF4A1* expressions in patients with HCC and healthy controls were detected and compared. Results showed that *SENP3-EIF4A1* was significantly reduced in HCC tissues and exosomes from the plasma of patients with HCC (*P*<0.05) and was primarily encapsulated by exosomes. The patients with HCC and the healthy controls could be distinguished using exosomal *SENP3-EIF4A1* (AUC=0.8028). The transfer of exosomal *SENP3-EIF4A1* secreted by normal cells to HCC cells stimulated apoptosis and weakened the invasion and migration abilities of HCC cells to suppress their malignant biological behavior (*P*<0.05). Additionally, exosomal *SENP3-EIF4A1* was capable of inhibiting tumor growth in vivo and modulating the expression of *ZFP36* by competitively binding to miR-9-5p. In conclusion, exosomal *SENP3-EIF4A1* is a new favorable biomarker for clinically detecting HCC, and *SENP3-EIF4A1* can be transmitted by exosomes from normal cells to HCC cells to inhibit the *in vitro* and *in vivo* development of HCC. Thus, exosomal *SENP3-EIF4A1* is involved in the communication between normal cells and HCC cells during the onset of HCC.

## INTRODUCTION

Hepatocellular carcinoma (HCC) is the most common malignant primary tumor in the liver [1]. The morbidity and mortality of HCC rank fifth and third, respectively, among all malignant tumors [2]. More than 600,000 people die each year from HCC [3]. The findings of epidemiological and experimental studies have demonstrated that hepatitis B and C virus infections, as well as drinking and smoking habits, may trigger and promote the development of HCC [4, 5]. Although great progress has been made in recent years through clinical treatment drugs and surgical treatment, the incidence and mortality of HCC remain high. HCC has become an important health issue for people all over the world. Thus, exploring the pathophysiological mechanism of the development of HCC and finding more effective targets for its prevention and treatment are urgent concerns.

Long non-coding RNAs (lncRNAs) are ubiquitously transcribed in the human genome [6]. LncRNAs exert indispensable effects on maintaining normal cell growth and function [7], and changes in lncRNA expression has a close relationship to cancer, such as pancreatic and breast cancers [8–10]. LncRNAs can act as competing endogenous RNAs (ceRNAs) to regulate mRNA activity and biological function by competitively binding to common microRNAs [11, 12]. They are also closely correlated with HCC, as reported in 2015 by Li C et al. [13] Xiong H et al. confirmed that lncRNA *HULC* stabilizes Sirt1 and weakens the chemosensitivity of HCC cells to induce autophagy [14]. Varying lncRNAs are involved in HCC development and progression, but the specific mechanism by which lncRNA *SENP3-EIF4A1* plays a role in HCC remains unclear.

As extracellular vesicles of endocytic origin, exosomes are secreted by different types of cells and considered to be extracellular messengers between tumor cells and their microenvironment because of their ability to transfer and exchange lncRNAs and other cargoes [15, 16]. Exosomal lncRNAs are clinical biomarkers in a stable state in the blood and can be used to diagnose tumors in individuals [17].

The present study examined exosomal lncRNAs in plasma samples from three patients with liver cancer and three healthy controls by using microarray sequencing techniques. The results indicated that *SENP3-EIF4A1*, which was remarkably decreased in the plasma of the patients with liver cancer, had the largest difference between the two groups (*P*=0.042). Subsequently, the expression of *SENP3-EIF4A1* in HCC tissues and exosomes was detected in the plasma samples. Our results revealed that *SENP3-EIF4A1* was remarkably decreased in HCC tissues and exosomes from the plasma of patients with HCC. Nevertheless, no data are currently available regarding the biological roles of exosomal *SENP3-EIF4A1* in HCC. The purpose of this study was to find a potential biomarker that could be used in the diagnosis of HCC and investigate whether exosomal *SENP3-EIF4A1* intervenes in cell-to-cell communication, which may result in the progression of HCC.

## RESULTS

### Characteristics of patients

The characteristics of the patients with HCC and the healthy controls are shown in Table 1. No differences in age, gender, and drinking status were found between the patients with HCC and the healthy controls (*P*>0.05).

**Table 1:** The characteristics of the hepatocellular carcinoma cases and healthy controls.

**Variables**	**Case (n=50)**	**Control (n=50)**	**P value**
Age(years) (mean ± SD)			0.8200
<50	14	12	
≥50	36	38	
Gender			0.6709
Male	32	35	
Female	18	15	
Drinking status			0.8153
Never	13	11	
Ever	37	39	
Tumor staging (TNM)			
I/ II	26	-	-
III/ IV	24	-	

Fisher's exact test for all variables between HCC cases and controls

### Expression of plasma exosomal *SENP3-EIF4A1* in patients with HCC

**Figure 1:**
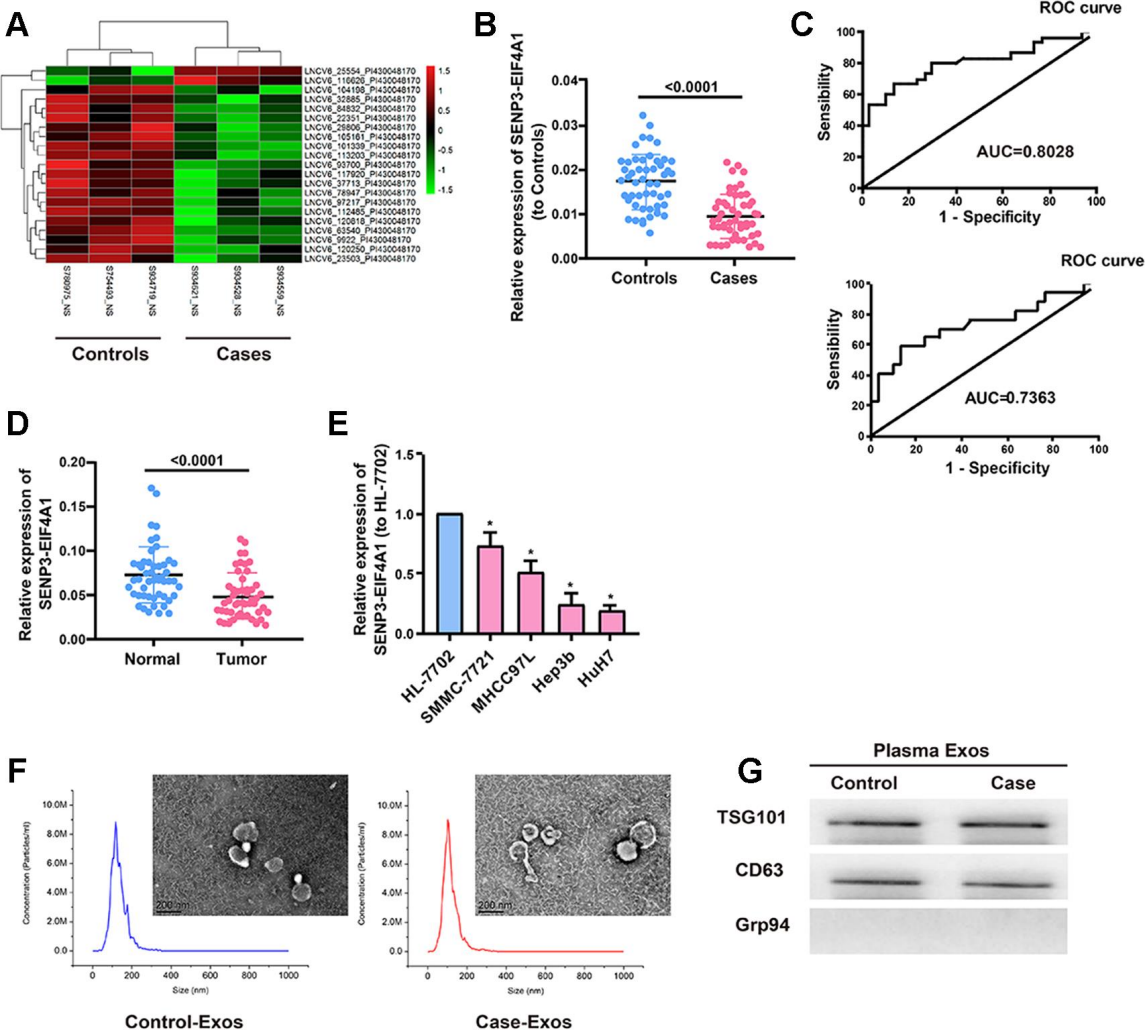
**Expression of plasma exosomal *SENP3-EIF4A1* in patients with HCC. Exosomes (exos) are isolated from the plasma of HCC patients and healthy controls.** (**A**) Heatmap illustrating the 21 differentially expressed lncRNAs between HCC patients’ plasma exos and healthy control patients’ plasma exos. (**B**) Detection of *SENP3-EIF4A1* in exos from plasma via qRT-PCR. (**C**) Receiver Operating Characteristic (ROC) curve for the *SENP3-EIF4A1* to distinguish HCC cases from controls (Top). Receiver Operating Characteristic (ROC) curve for the *SENP3-EIF4A1* to distinguish TNM I/II stage HCC cases from controls (bottom). (**D**) Determination of *SENP3-EIF4A1* in tumor tissues and adjacent normal tissues via qRT-PCR. (**E**) *SENP3-EIF4A1* is lowly expressed in HCC cells compared with HL-7702 cell line. (**F**) Micrographs and the size distribution of exos isolated from the plasma of HCC patients (left) and healthy controls (right) were detected using TEM (bars =200 nm) and NTA. (**G**) Detection of TSG101, CD63 and Grp94 in circulating exos via Western blotting. Results are shown as mean ± SD. *P<0.05. All of the experiments were performed in triplicate.

We used microarray sequencing techniques to examine the exosomal lncRNAs in the plasma samples extracted from the three patients with liver cancer and the three healthy controls. Results revealed that *SENP3-EIF4A1*, which was significantly reduced in the plasma of patients with liver cancer, had the largest difference between the two groups (*P*=0.042, Figure 1A). Subsequently, the expression of *SENP3-EIF4A1* in plasma exosomes was examined. Results revealed that exosomes from the patients with HCC had a significantly decreased expression of *SENP3-EIF4A1* than did exosomes from the healthy controls (*P*<0.05, Figure 1B). The potential effect of exosomal *SENP3-EIF4A1,* a noninvasive biomarker, was also assessed by generating receiver operating characteristic (ROC) curves. As shown in Figure 1C, the area under the curve (AUC) of exosomal *SENP3-EIF4A1* was 0.8028. We classified the patients with HCC according to the TNM stage, and tested whether SENP3-EIF4A1 could distinguish early HCC. The AUC of exosomal *SENP3-EIF4A1* was 0.7363, as exhibited in Figure 1C. Ultimately, the content of *SENP3-EIF4A1* in liver cancer tissues and adjuvant normal tissue verified that its expression was substantially reduced in HCC tumor tissues (Figure 1D). Similarly, *SENP3-EIF4A1* was lowly expressed in HCC cells compared with the HL-7702 cell line (Figure 1E). The HCC tumor tissues were then divided into high (n=25) and low groups (n=25) according to the median expression value of *SENP3-EIF4A1*. *SENP3-EIF4A1* was found to be associated with Tumor size, Tumor stage and Lymph node metastasis but not with other clinicopathologic features (Table 2).

**Table 2:** Clinicopathologic correlation of lncRNA SENP3-EIF4A1 expression in HCC tissues.

**Feathers**	**Number**	**High**	**Low**	**P value**
All cases	50	25	25	
Age(years)				0.7536
< 50	14	6	8	
≥50	36	19	17	
Gender				0.7688
Male	32	15	17	
Female	18	10	8	
Drinking status				0.5202
Never	13	8	5	
Ever	37	17	20	
Tumor size (cm)				0.0421
< 5	30	19	11	
≥ 5	20	6	14	
Tumor stage (TNM)				0.0465
I/ II	26	17	9	
III/ IV	24	8	16	
Lymph node metastasis				0.0378
Yes	18	5	13	
No	32	20	12	

Total data from 50 HCC patients were analyzed. For the expression of SENP3-EIF4A1 was assayed by qRT-PCR, the median expression level was used as the cutoff. Data were analyzed by Fisher's exact test. P-value in bold indicates statistically significant.

Subsequently, transmission electron microscopy (TEM) and nanoparticle tracking analysis were carried out to analyze the characteristics of the plasma exosomes from the patients with HCC and the healthy controls. Results showed that exosomes from the patients with HCC had the same sizes as those from the healthy controls (Figure 1F). Western blot results showed that TSG101 and CD63 (exosome markers) existed rather than the negative internal reference Grp94 (Figure 1G). These findings suggest that exosomal *SENP3-EIF4A1* may be a favorable biomarker to distinguish patients with HCC from healthy controls.

### Effect of *SENP3-EIF4A1* on HCC cellular phenotype

**Figure 2:**
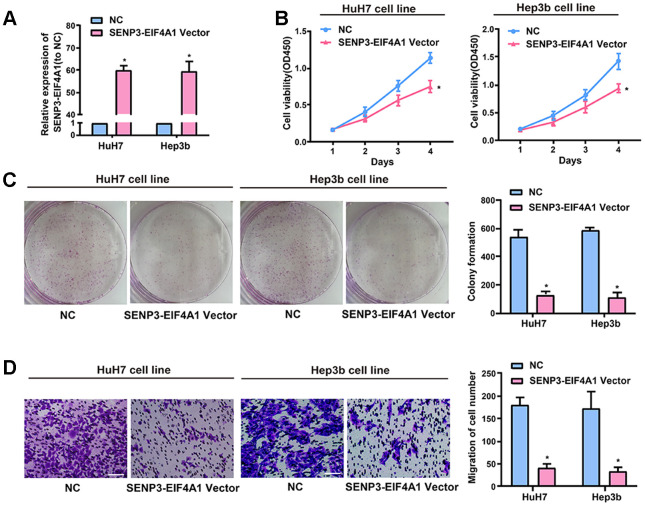
**Effect of *SENP3-EIF4A1* on HCC cellular phenotype. HuH7 and Hep3b cells are transfected with *SENP3-EIF4A1* plasmids or NC vectors.** (**A**) Detection of the mRNA level of *SENP3-EIF4A1* via qRT-PCR. (**B**) Examination of cell viability via CCK8 assays. (**C**) Determination of cell colony formation ability via a colony-forming growth assay. The colonies are counted and captured. (**D**) Representative images of migration assays of HuH7 and Hep3b cells (bars =100 μm). The cells are counted. Results are shown as mean ± SD. *P<0.05. All of the experiments were performed in triplicate.

This study explored the biological role of *SENP3-EIF4A1* *in vitro* in light of the downregulated expression of *SENP3-EIF4A1* in the tissues and exosomes from the plasma of patients with HCC. HuH7 and Hep3b cells were transfected with *SENP3-EIF4A1* overexpression plasmids or NC vectors, and the expression of *SENP3-EIF4A1* (Figure 2A) was determined via quantitative real-time polymerase chain reaction (qRT-PCR). The results illustrated that the proliferation of HuH7 and Hep3b cells transfected with *SENP3-EIF4A1* overexpression for 24 h was evidently inhibited compared to the cells transfected with NC vectors (Figure 2B). These results are consistent with the results of colony formation assays (Figure 2C). Moreover, the migration ability of the two cell lines transfected with *SENP3-EIF4A1* overexpression plasmids for 24 h was obviously suppressed (Figure 2D). Meanwhile, the knockdown of *SENP3-EIF4A1* in SMMC-7721 cells promoted cell proliferation, colony formation, and migration (Supplementary Figure 1).

### Exosomal *SENP3-EIF4A1* mediates intercellular communication

**Figure 3:**
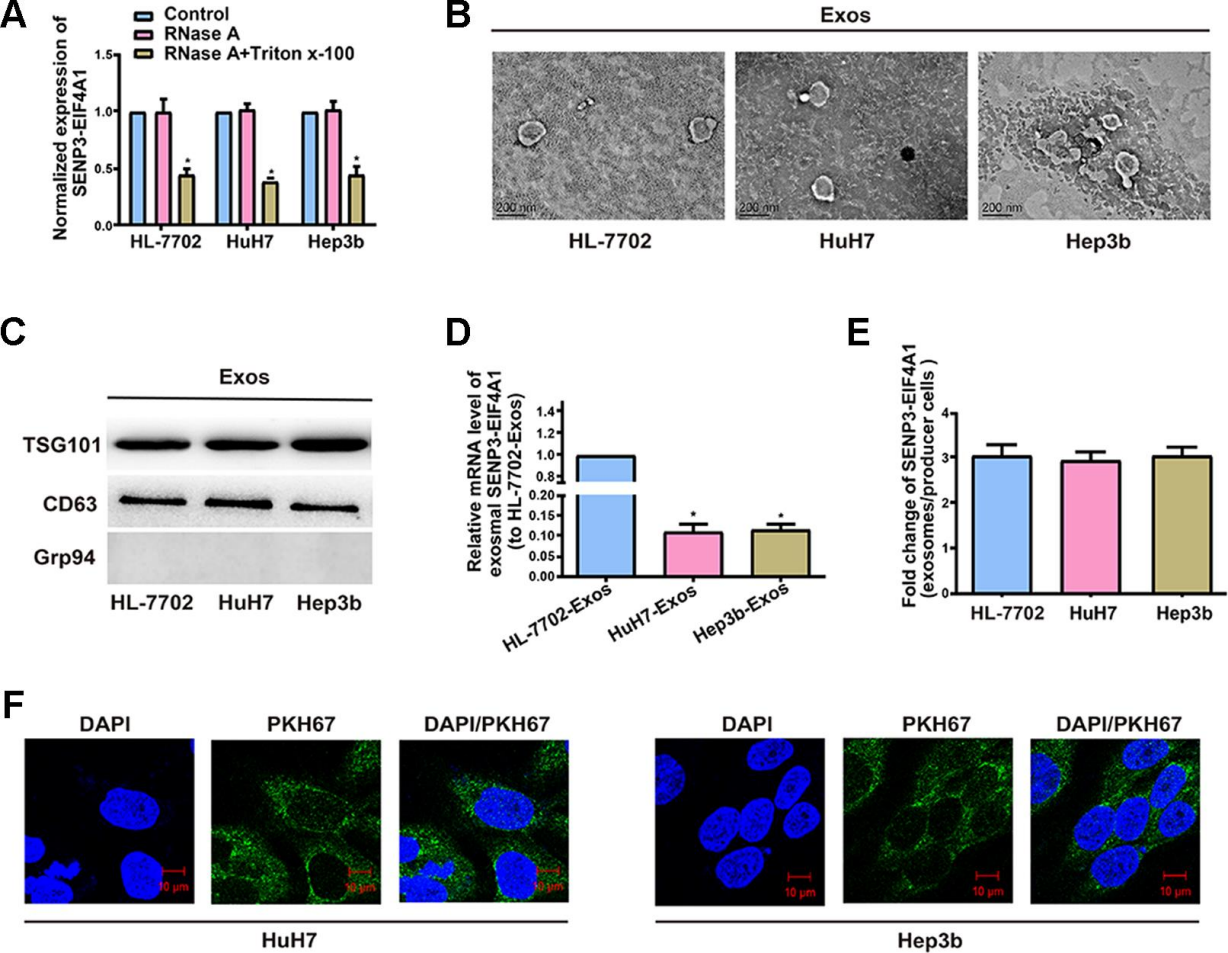
**Exosomal *SENP3-EIF4A1* mediates intercellular communication. Exos are separated from the medium of HL-7702, Hep3b and HuH7 cells.** (**A**) Detection of the normalized expression of *SENP3-EIF4A1* in the medium of HL-7702, Hep3b and HuH7 cells receiving treatment with RNase (2μg/ml) alone or combined treatment with RNase (2μg/ml) and Triton X-100 (0.1%) for 20min via qRT-PCR. (**B**) Micrographs of exos separated from HL-7702 (left), HuH7 (middle) and Hep3b cells (right, bars=200 nm). (**C**) Examination of TSG101, CD63 and Grp94 in exos of cell lines via Western blotting. (**D**) Detection of exosomal *SENP3-EIF4A1* of HL-7702, Hep3b and HuH7 via qRT-PCR. (**E**) Assessment of the fold change of SENP3-EIF4A1 between exos of HL-7702, Hep3b and HuH7 and their producer cells via qRT-PCR. (**F**) Exos from HL-7702 cells are labeled with PKH67; green represents PKH67, and blue represents nuclear DNAs stained by DAPI. Hep3b and HuH7 cells undergo 3 h of incubation with exos from HL-7702 cells. Results are shown as mean ± SD. *P<0.05. All of the experiments were performed in triplicate.

The existing pattern of extracellular *SENP3-EIF4A1* was investigated. RNase treatment did not change the level of *SENP3-EIF4A1* in the medium, but the combined treatment of RNase and TritonX-100 notably decreased this level (Figure 3A). This finding indicated that extracellular *SENP3-EIF4A1* is packaged by a membrane and cannot be directly released. TEM was adopted to determine the sizes of the exosomes (Figure 3B). The presence of TSG101 and CD63 and the absence of negative internal reference Grp94 were verified via Western blot analysis (Figure 3C). Exosomal *SENP3-EIF4A1* exhibited a remarkably higher expression in HL-7702 cells than in Hep3b and HuH7 cells (Figure 3D). *SENP3-EIF4A1* levels in exosomes were likewise increased approximately three times in comparison with those in producer cells (Figure 3E). Hence, the level of *SENP3-EIF4A1* was larger in exosomes from HL-7702 cells than in exosomes from Hep3b and HuH7 cells. In subsequent procedures, a green fluorescent marker, PKH67, was utilized to label exosomes from HL-7702 cells and then the labeled exosomes were applied to incubate recipient cells (Hep3b and HuH7 cells) for 3 h. PKH67 was found to be present in the cytoplasm of the recipient cells (Figure 3F).

### Effect of exosomal *SENP3-EIF4A1* on HCC cellular phenotype

**Figure 4:**
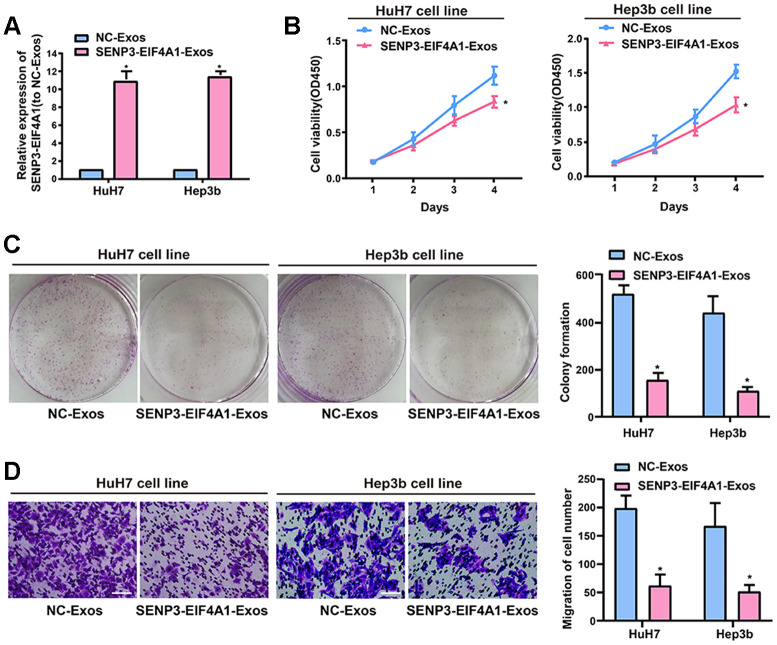
**Effect of exosomal *SENP3-EIF4A1* on HCC cellular phenotype.** Exosomes separated from HL-7702 cells transfected with *SENP3-EIF4A1* overexpression plasmids or NC vectors are named *SENP3-EIF4A1*-Exos or NC-Exos, respectively. After extraction, their exosomes are added to the HuH7 and Hep3b cells for 24h. (**A**) Detection of the mRNA level of *SENP3-EIF4A1* via qRT-PCR. (**B**) Detection of cell viability by a CCK8 assay. (**C**) Examination of cell colony formation ability by a colony-forming growth assay. The colonies are counted and captured. (**D**) Representative images of migration assays of HuH7 and Hep3b cells (bars =100 μm). The cells are counted. Results are shown as mean ± SD. *P<0.05. All of the experiments were performed in triplicate.

Exosomes exert crucial effects on intercellular communication such that the physiological function of the recipient cells is changed by bioactive factors, including lncRNAs [18]. The silencing of *SENP3-EIF4A1* frequently occurs in patients with HCC and HCC cell lines but not in healthy controls. Thus, *SENP3-EIF4A1* can be a tumor suppressor. The study results showed that exosomes were capable of transmitting *SENP3-EIF4A1* from HL-7702 cells into HuH7 and Hep3b cells. Thus, we speculated that the biological functions of the HuH7 and Hep3b cells could be influenced by exosomal *SENP3-EIF4A1* from HL-7702 cells. However, exosomes carry a variety of cargoes, including transcriptional regulators, varying RNA species, DNAs, and lipids [19]. Thus, exosomes from HL-7702 cells transfected with *SENP3-EIF4A1* overexpression plasmids or NC vectors, namely, *SENP3-EIF4A1*-Exos and NC-Exos, respectively, were separated to determine the functions of exosomal *SENP3-EIF4A1*. Thereafter, *SENP3-EIF4A1*-Exos or NC-Exos (100 μg/ml) were added to HuH7 and Hep3b cells for 24 h. The results revealed that the levels of *SENP3-EIF4A1* in HuH7 and Hep3b cells added with *SENP3-EIF4A1*-Exos were prominently higher than those in HuH7 and Hep3b cells added with NC-Exos for 24 h (Figure 4A). *SENP3-EIF4A1*-Exos could evidently suppress the proliferation of HuH7 and Hep3b cells as well (Figure 4B). Additionally, similar results were obtained through colony formation assays (Figure 4C). *SENP3-EIF4A1*-Exos was also able to inhibit the migration of HuH7 and Hep3b cells (Figure 4D). The exosomes separated from HL-7702 cells transfected with *SENP3-EIF4A1* siRNA or NC siRNA are named *SENP3-EIF4A1* siRNA-Exos and NC siRNA-Exos, respectively. Findings indicated that *SENP3-EIF4A1* siRNA-Exos promoted the proliferation, colony formation, and migration of SMMC-7721 cells (Supplementary Figure 2).

### Subcellular distribution of *SENP3-EIF4A1*

**Figure 5:**
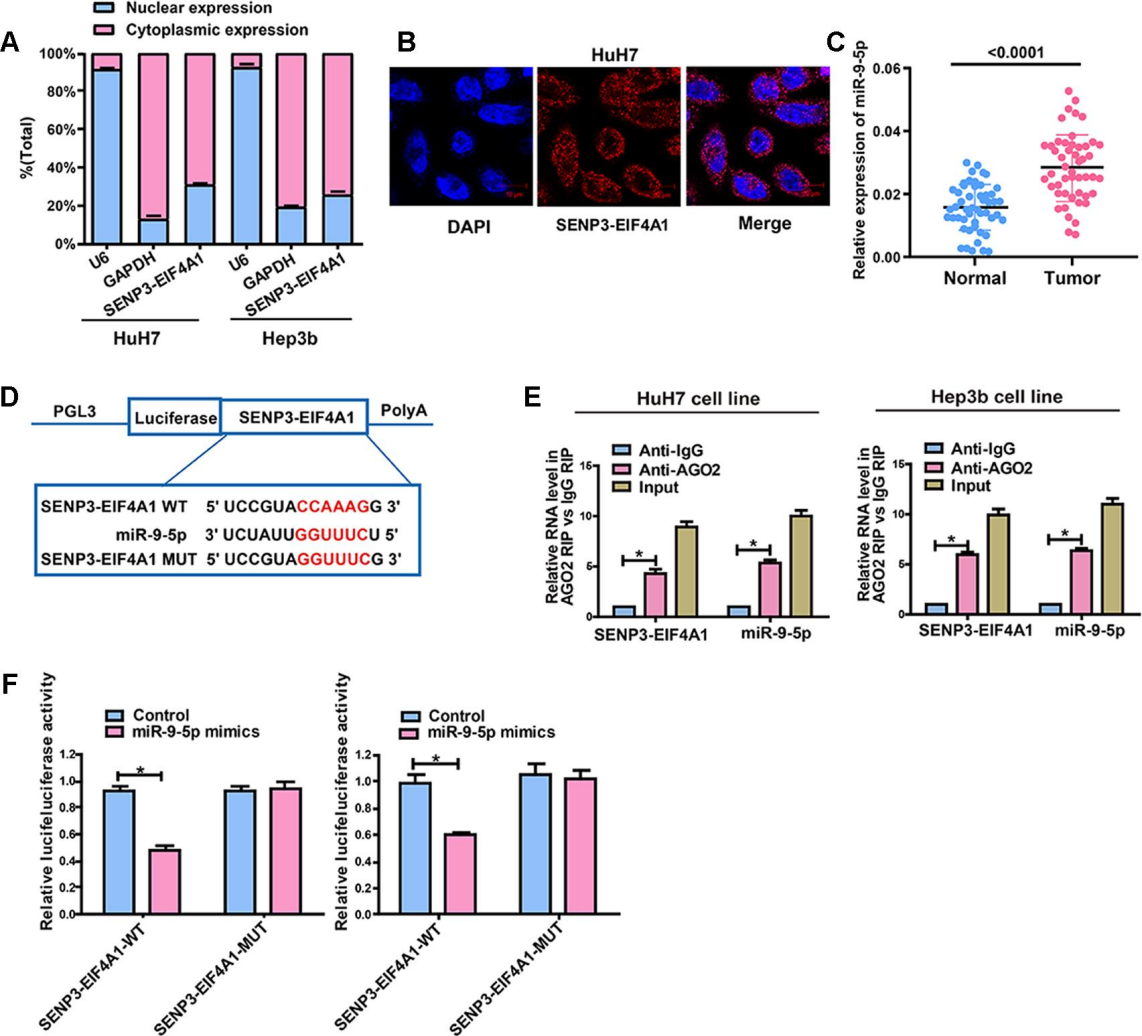
***SENP3-EIF4A1* interacts with miR-9-5p in a direct manner.** (**A**) Cytoplasmic and nuclear levels of *SENP3-EIF4A1* in HuH7 and Hep3b cells analyzed by qRT-PCR. (**B**) The RNA-FISH results verified that *SENP3-EIF4A1* was distributed mostly in the cytoplasm in HuH7 cells. (**C**) MiR-9-5p expression in HCC tissues and adjacent normal tissues detected by qRT-PCR. (**D**) Bioinformatics evidence of binding of miR-9-5p onto 3'-UTR of *SENP3-EIF4A1*. (**E**) RIP experiments for the amount of *SENP3-EIF4A1* and miR-9-5p in HuH7 and Hep3b cells. (**F**) Dual-luciferase reporter gene assay in HuH7 and Hep3b cells after transfection with negative control or miR-9-5p mimics, Renilla luciferase vector pRL-SV40 and the reporter constructs. Data are presented as mean ± SD. *P<0.05. All of the experiments were performed in triplicate.

The subcellular distribution of lncRNA determines its biological function. HCC cells were isolated into cytoplasmic and nuclear fractions with GAPDH and U6 as controls, respectively, to confirm the cellular localization of *SENP3-EIF4A1*. The qRT-PCR results showed that 69.1% and 74.2% of *SENP3-EIF4A1* were distributed in the cytoplasmic fraction of HuH7 and Hep3b cells, respectively (Figure 5A). The subcellular localization of *SENP3-EIF4A1* was verified using RNA fluorescence in situ hybridization (FISH). The results showed that most of the positives were oriented in the cytoplasm, and the minority was in the nucleus of HuH7 cells (Figure 5B). Thus, *SENP3-EIF4A1* likely participated in the development of HCC through post-transcriptional regulation.

### *SENP3-EIF4A1* is targeted by miR-9-5p

Given that *SENP3-EIF4A1* is primarily located in the cytoplasmic fraction, we hypothesized that *SENP3-EIF4A1* may act as a ceRNA in the development of HCC. The data from qRT-PCR revealed that miR-9-5p expression was higher while *SENP3-EIF4A1* expression was lower in HCC tumor tissues compared with normal tissues (Figure 5C). RegRNA and Starbase prediction indicated that sequences in miR-9-5p highly matched the 3′ untranslated region of *SENP3-EIF4A1*. We established pGL3-*SENP3-EIF4A1*-WT and pGL3-*SENP3-EIF4A1*-MUT on the basis of these binding sequences (Figure 5D). RNA immunoprecipitation analysis was carried out to elucidate whether *SENP3-EIF4A1* was involved in the RNA-containing ribonucleoprotein complex. The qRT-PCR results showed that *SENP3-EIF4A1* was enriched in the anti-Ago2 antibody than the controls. Similar results were yielded in miR-9-5p (Figure 5E). Luciferase activity was obviously downregulated in HuH7 and Hep3b cells co-transfected with wild-type (WT) *SENP3-EIF4A1* and miR-9-5p mimics but was not changed in those transfected with mutant (MUT) *SENP3-EIF4A1* (Figure 5F). This outcome suggested that miR-9-5p can bind to *SENP3-EIF4A1* *in vitro*.

### *SENP3-EIF4A1* regulates *ZFP36*, the target gene of miR-9-5p

**Figure 6:**
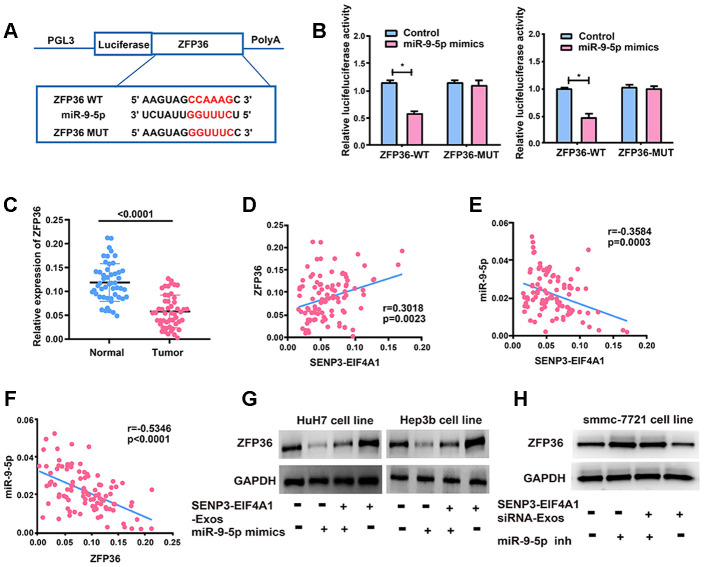
***ZFP36* is the direct target of miR-9-5p.** (**A**) The putative miRNA binding sites in the *ZFP36* sequence. (**B**) Direct target sites verified by the dual-luciferase reporter gene assay. (**C**) *ZFP36* expression in HCC tissues and adjacent normal tissues detected via qRT-PCR. (**D**) Bivariate correlation analysis of the relationship between *SENP3-EIF4A1* and *ZFP36* expression level. (**E**) There was a significantly negative correlation between the expression level of *SENP3-EIF4A1* and the expression level of miR-9-5p in the same paired HCC samples. (**F**) There was a significantly negative correlation between the expression level of *ZFP36* and the expression level of miR-9-5p in the same paired HCC samples. (**G**) ZFP36 in HuH7 and Hep3b cells with *SENP3-EIF4A1*-Exos and/or miR-9-5p mimics examined via Western blotting. (**H**) ZFP36 in SMMC-7721 cells with *SENP3-EIF4A1* siRNA-Exos and/or miR-9-5p inhibitor examined via Western blotting. Data are shown as mean ± SD. *P<0.05. All of the experiments were performed in triplicate.

The target genes of miR-9-5p were screened out by bioinformatics prediction (TargetScan, Starbase, and RegRNA) to investigate the potential role of miR-9-5p in HCC development. Finally, *ZFP36* was selected for further analyses. Luciferase plasmids pGL3-*ZFP36*-WT and pGL3-*ZFP36*-MUT were constructed and co-transfected with miR-9-5p mimics or NC in HuH7 and Hep3b cells, respectively (Figure 6A). The luciferase activity of the WT reporter group was inhibited, whereas that of the MUT reporter group did not change (Figure 6B). This finding suggested that *ZFP36* is the potential target gene of miR-9-5p. Subsequently, *ZFP36* expression in HCC cell lines was examined via qRT-PCR, and results demonstrated that the mRNA levels of *ZFP36* were remarkably inhibited in HCC tumor tissues (Figure 6C). Bivariate correlation analysis was also performed to assess the interactions of *SENP3-EIF4A1* with miR-9-5p and *ZFP36* in the tissues at the mRNA level (Figures 6D–6F). According to the analysis, *SENP3-EIF4A1* was positively correlated with the expression levels of *ZFP36*. Meanwhile, miR-9-5p was inversely correlated with *SENP3-EIF4A1* and *ZFP36* expression in HCC tissues. Figure 6G shows that miR-9-5p mimics lowered the expression of *ZFP36*, which was reversed by exosomal *SENP3-EIF4A1*. As shown in Figure 6H, miR-9-5p inhibitor promoted the expression of *ZFP36*, which was reversed by *SENP3-EIF4A1* siRNA-Exos.

### Effects of *SENP3-EIF4A1* overexpression and exosomal *SENP3-EIF4A1* on tumor *in vivo*

**Figure 7:**
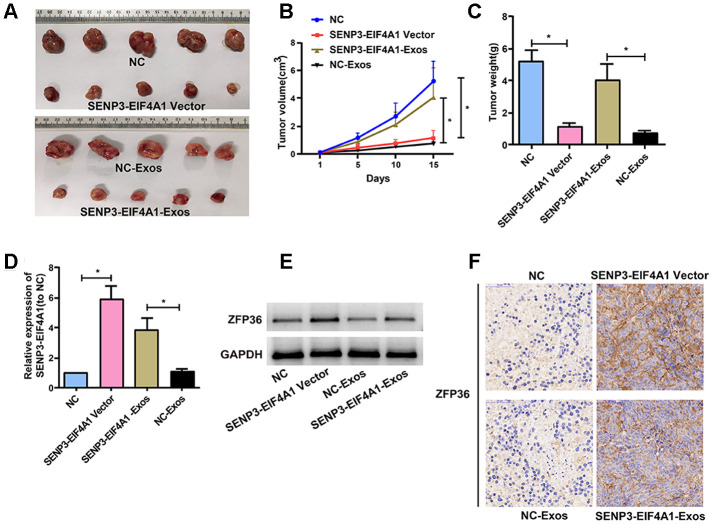
**Effects of *SENP3-EIF4A1* overexpression and exosomal SENP3-EIF4A1 on tumor *in vivo*.** HuH7 cells are transfected with *SENP3-EIF4A1*/NC lentiviral vectors, namely *SENP3-EIF4A1* vectors and NC, respectively. Exos are separated from HL-7702 cells transfected with *SENP3-EIF4A1*/NC lentiviral vectors, namely *SENP3-EIF4A1*-Exos and NC-Exos, respectively. (**A**) The xenografts from nude mice inoculated in NC, *SENP3-EIF4A1*-Exos, *SENP3-EIF4A1* vectors and NC-Exos (n=5, each group). (**B**) The tumor volumes are measured every other day after injection. (**C**) Determination of the tumor weights in nude mice after 15 days. (**D**) Detection of *SENP3-EIF4A1* expression in tumor tissues of nude mice treated with NC, *SENP3-EIF4A1* vectors, *SENP3-EIF4A1*-Exos and NC-Exos by qRT-PCR. (**E**) Detection of ZFP36 in tumor tissues via Western blotting. (**F**) Analysis of ZFP36 expression in tumor tissues by IHC. Results are shown as mean ± SD. *P<0.05.

HuH7 and HL-7702 cells transfected with *SENP3-EIF4A1*/NC lentiviral vectors or *SENP3-EIF4A1-*Exos/NC-Exos were injected into nude mice to further explore the effects of *SENP3-EIF4A1* overexpression and exosomal *SENP3-EIF4A1* on HCC in vivo. The results denoted that mean tumor weight and volume were evidently reduced in the *SENP3-EIF4A1* overexpression group compared with those in the NC group (Figure 7A–7C). This result was consistent with those of the *in vitro* analysis. As shown in Figures 7A–7C, the size and weight of the tumor tissues of nude mice injected with *SENP3-EIF4A1*-Exos were also decreased. The expression of *SENP3-EIF4A1* in the tumor tissues of mice transfected with *SENP3-EIF4A1* vectors or *SENP3-EIF4A1*-Exos was elevated (Figure 7D). Moreover, Western blot and immunohistochemistry was carried out to explore *ZFP36* expression. Figures 7E and 7F show that the level of *ZFP36* expression was notably elevated in the models of *SENP3- EIF4A1* overexpression and *SENP3-EIF4A1*-Exos, respectively.

## DISCUSSION

HCC is the fifth most common tumor in patients around the world and ranks third following lung cancer and gastric cancer among lethal cancers [20]. The results of this study revealed that *SENP3-EIF4A1* was decreased in HCC tissues and plasma. In addition, the exosomal *SENP3-EIF4A1* secreted by normal cells was transferred to HCC cells, where it prominently impeded the malignant behavior of HCC cells by reducing the proliferation and migration abilities of cancer cells and the growth of tumor *in vivo*. Therefore, exosomal *SENP3-EIF4A1* can be a potential biomaker for HCC detection. Moreover, exosomal *SENP3-EIF4A1* sponged miR-9-5p to protect *ZFP36* and inhibit the biologically malignant behavior of HCC.

The expression of dysregulated lncRNAs was observed in a variety of cancers, including HCC [21, 22]. The physiological condition of the donor cells can be reflected by exosomal lncRNAs, and these lncRNAs can be captured by recipient cells to induce a train of cellular responses [18, 23, 24]. Exosomal lncRNAs are also potential cancer biomarkers for patients with different cancers [25–27] and are beneficial for cancer identification.

The findings indicated that *SENP3-EIF4A1* was primarily transferred by exosomes from cells. In-depth investigations have been conducted regarding the roles of exosomal lncRNAs in cancers [28, 29]. For instance, exosomes from normal cells transfer *PTENP1* to bladder cells to block the progression of bladder cancer [30]. The levels of *SENP3-EIF4A1* were found to be elevated in normal cells and their exosomes, and its expression in exosomes showed an almost threefold elevation compared with that in producer cells. Fluorescence microscopy was then carried out, and the results revealed that PKH67-labeled exosomes from normal cells could be transferred into HCC cells. These findings implied that HL-7702 cells probably secrete exosomal *SENP3-EIF4A1* that can be transferred to the surrounding HCC cells. The transfer of exosomal lncRNAs secreted from cancer cells into normal cells has been widely investigated, but reports regarding exosomal lncRNAs transferred from normal cells to cancer cells have been rare. The results of this study displayed that *SENP3-EIF4A1*-Exos from HL-7702 cells stimulated *SENP3-EIF4A1* expression and impeded the migration and proliferation of HCC cells. Exosomal *SENP3-EIF4A1* also exerted an inhibitory effect on tumor growth *in vivo*, which is consistent with previous findings *in vitro* in this study. The findings mentioned above imply that *SENP3-EIF4A1* is transferred from normal cells to HCC cells via exosomes in a direct manner and modulates the biological functions of HCC *in vitro* and *in vivo*.

*SENP3-EIF4A1* is the lncRNA located at 17p13.1. The qRT-PCR results showed that *SENP3-EIF4A1* is mainly distributed in the cytoplasmic fraction of HCC cells. We may conclude that *SENP3-EIF4A1* participates in the development of HCC through post-transcriptional regulation by acting as a ceRNA involved in the tumorigenesis of HCC. Our findings suggested that exosomal *SENP3-EIF4A1* compete for miR-9-5p to modulate *ZFP36* levels.

There remain insufficiencies in this research. The role of *SENP3-EIF4A1* siRNA-Exos should be further explored *in vivo*. Furthermore, a corresponding subcutaneous tumorigenesis experiment should be performed in *SENP3-EIF4A1*-Exos + miR-9-5p mimics/*SENP3-EIF4A1*-Exos + *ZFP36* shRNA rescue groups to verify the ceRNA mechanism of *SENP3-EIF4A1*/miR-9-5p/*ZFP36*.

The results of this study suggest that exosomal *SENP3-EIF4A1* can be a new valuable biomarker for diagnosing HCC. Additionally, exosomal *SENP3-EIF4A1* in normal cells can be transferred to HCC cells, and exogenous *SENP3-EIF4A1* alleviates the malignant phenotype of HCC cells in vitro and *in vivo*. Exosomal *SENP3-EIF4A1* may likewise induce miR-9-5p to modulate *ZFP36* and block HCC progression, that is, exosomal *SENP3-EIF4A1* mediates intercellular communication during the carcinogenesis of HCC.

## MATERIALS AND METHODS

### Research design and subjects

Written informed consent was obtained from all subjects, and this study scheme obtained the approval of the institutional review board of the Affiliated Hospital of Youjiang Medical College for Nationalities. In this research, we analyzed plasma samples from 50 patients with HCC and 50 healthy controls and 50 pairs of tumor and adjacent normal tissues obtained from patients with HCC from the Affiliated Hospital of Youjiang Medical College for Nationalities. The study was approved by the ethics committee of the Affiliated Hospital of Youjiang Medical College for Nationalities, and informed consent for the research use of clinical information and tissues was signed by the subjects involved.

### Cell lines

One human liver normal cell line (HL-7702) and four HCC cell lines (SMMC-7721, MHCC97L, HuH7, and Hep3b) were bought from the Cell Bank of the Typical Culture Preservation Committee of the Chinese Academy of Sciences (Shanghai, China) and kept in an RPMI-1640 medium (Gibco BRL, Rockville, Maryland, USA) with 10% fetal bovine serum (FBS, Gibco BRL, Rockville, Maryland, USA) under 5% CO_2_ at 37 °C.

### Separation of exosomes

The collected plasma and culture medium were centrifuged at 3000 × g for 15 min to discard cells and cellular debris. Next, Exoquick exosome precipitation solution (System Biosciences) was applied to separate exosomes.

### Nanoparticle tracking analysis (NTA)

We measured the exosome particle size and concentration using NTA with ZetaView PMX 110 (Particle Metrix, Meerbusch, Germany). NTA measurement was recorded and analyzed at 11 positions. The ZetaView system was calibrated using 110 nm polystyrene particles.

### Transmission electron microscopy (TEM)

Exosomes were suspended in 100 μl of PBS, fixed with 5% glutaraldehyde at incubation temperature and preserved at 4 °C for TEM analysis. A drop of exosome sample was then placed on a copper grid coated with carbon and immersed in a 2% phosphotungstic acid solution (pH 7.0) for 30 s in accordance with the steps for TEM sample preparation. Finally, a transmission electron microscope (TecnaiG2 Spirit Bio TWIN, FEI, USA) was utilized to observe the preparations.

### RNA isolation and qRT-PCR analysis

The separation of total RNAs from tissues and cell lines was achieved by using TRIzol reagent (Invitrogen, CA, USA). ExoRNeasy Midi Kit (Qiagen, Valencia, CA, USA) was utilized for exosomal RNA extraction from plasma and culture medium according to the manufacturer’s instructions. cDNA synthesis was performed using a high-capacity cDNA reverse transcription kit (Thermo Fisher Scientific, Vilnius, Lithuania). Subsequently, an ABI 7900 system (Applied Biosystems, CA, USA) and SYBR Green assays (TaKaRa Biotechnology, Dalian, China) were adopted for qRT-PCR. LncRNA expression level was normalized with GAPDH as internal control, and 2^−ΔCt^ method was adopted to assess the fold change in lncRNA expression. The primer sequences used were as follows: SENP3-EIF4A1: F 5′ CCGCCAGTTCTACATCAACG 3′, R 5′ TTCCTCCGGGTGTTGATGAA 3′, GAPDH: F 5′ CCGGGAAACTGTGGCGTGATGG 3′, R 5′ AGG TGGAGGAGTGGGTGTCGCTGTT 3′, ZFP36: F 5′ GACTGAGCTATGTCGGACCTT 3′, R 5′ GAGT TCCGTCTTGTATTTGGGG 3′.

### Exosome labeling

Exosomes from cells (1.5 × 10^6^) were suspended in 100 μl of PBS containing 1 mL of PKH67 (Sigma, mixed in Diluent C) and incubated at room temperature for 4 min. Afterward, exosome labeling was stopped through the addition of 2 ml of 0.5% bovine serum albumin, and Exoquick exosome precipitation solution was used to separate the exosomes that were stained. The exosomes were placed into 9.6 ml of basic media for suspension, 250 μl of which was added to the sub-confluent layer of HuH7 and Hep3b cells. Subsequently, the cells were subjected to 3 h of incubation at 37 °C, rinsed, and then fixed at room temperature. Finally, the nuclei were stained with 4′, 6-diaminido-2-phenylindole (Sigma) for 10 min and observed under a fluorescence microscope (Zeiss, LSM700B, Germany).

### Cell transfection

The *SENP3-EIF4A1* overexpression plasmids, siRNA, empty vector (NC), miR-9-5p inhibitor, miR-9-5p mimics, and lentiviral vectors with *SENP3-EIF4A1*/NC were synthesized by GeneChem (Shanghai, China). The cells were transfected with a Lipofectamine 2000 (Invitrogen, Carlsbad, CA, USA) transfection reagent according to the manufacturer’s instructions.

### Cell proliferation assay

Approximately 4.0 × 10^3^ HuH7 and Hep3b cells transfected with *SENP3-EIF4A1* overexpression plasmids/NC vectors or incubated with exosomes were placed in 96-well plates. Cell counting kit 8 (Dojindo Laboratories, Kumamoto, Japan) was utilized for cell proliferation with reference to the manufacturer’s protocol. The absorbance was evaluated at 450 nm with an Infinite M200 spectrophotometer (Tecan, Switzerland). All experiments were performed in triplicate.

### Colony formation assays

After 24 h of transfection or exosome incubation, about 200 cells were placed into the 96-well plate and kept in RPMI-1640 with 10% FBS. The medium was replaced every 4 days. Two weeks later, the colonies were fixed with 95% methanol and stained with 0.1% crystal violet. All experiments were performed in triplicate.

### Cell migration assays

The Transwell system was also used to determine cell migration. HCC cells were inoculated into the upper surface of the chamber. The cells on the lower surface of the chamber were incubated for 24 h at 37 °C, mixed with methanol, and stained with 0.1% crystal violet. The cells that did not migrate were wiped with a cotton swab. Five randomly selected fields were photographed and counted per well. Each experiment was performed in triplicate.

### Animal experiments

The HuH7 cell lines (1 × 10^7^ cells in 0.1 ml of PBS) were stably transfected with SENP3-EIF4A1/ NC lentiviral vector and then injected subcutaneously into the right flank of male nude mice (5 weeks old, 5 mice each group). After tumors grew to 100 mm^3^, tumor size was examined every two days. The mice were sacrificed after two weeks, and tumor weight was measured. To investigate the effect of exosomal SENP3-EIF4A1 *in vivo*, we only injected HuH7 cells subcutaneously into the right flank of male nude mice, when tumors grew to 100 mm^3^, isolated different exosomes (10 μg) from HL-7702 cells transfected with SENP3-EIF4A1/ NC lentiviral vector were then injected into the center of tumor every two days. All surgeries were performed under sodium pentobarbital anesthesia via intraperitoneal injection (40 mg/kg) and all efforts were made to minimize suffering. Finally, all the mice were euthanized two weeks later, and the tumor weights were measured. The animal experiment obtained the approval of the Institutional Animal Care and Use Committee of Affiliated Hospital of Youjiang Medical College for Nationalities, and experiments were performed following the NIH guidelines on animal welfare.

### Immunohistochemistry (IHC)

Representative areas were selected via H&E staining. Anti-ZFP36 (Abcam, Shanghai, China) was applied for IHC based on the manufacturer's protocol.

### Subcellular fractionation location

After the number of cells reached 1×10^6^, the cells were fully lysed with 200μl Lysis Buffer J added in the culture flask. Through centrifugation, cytoplasmic RNAs appeared in the supernatant and nuclear RNAs in the remaining liquid. Then the supernatant was removed, the liquid with cytoplasmic RNAs and nuclear RNAs were added with Buffer SK and absolute ethyl alcohol, respectively, and column centrifugation was carried out to elute the two types of RNAs, after which they were taken separately for qRT-PCR to determine the cellular localization of *SENP3-EIF4A1*.

### RNA FISH

FISH assay was performed using Ribo™ Fluorescent in Situ Hybridization Kit (Ribobio Company, China). *SENP3-EIF4A1* probe was designed and synthesized by Ribobio Company and labeled with Cy3 fluorescent dye. RNA FISH were performed using fluorescent in situ hybridization kit (RiboBio) following the manufacturer’s instructions. Fluorescence detection was performed with a confocal laser-scanning microscope (Leica, Germany).

### Dual-luciferase reporter assay

After seeded into the 24-well-plate, cells (1×10^4^) were then co-transfected with luciferase reporters (10ng) and miR-9-5p mimics (80nM) or NC using Lipofectamine 2000 (Invitrogen, Shanghai, China). The Dual-Luciferase Reporter Assay System (Promega, Shanghai, China) was utilized to perform the luciferase reporter assay.

### RNA binding protein immunoprecipitation (RIP) assay

RIP assay was conducted in strict accordance with the instructions of the Millipore kit (Millipore, Bedford, MA, USA). After the cells were lysed, the detection antibody (8μg per reaction system) was added for incubation at 4°C overnight, and it was reheated to room temperature for 1h. The complex was captured using protein G magnetic beads, and the buffer was washed to extract RNAs. The extracted RNAs were then reversely transcribed and their levels were detected by qRT-PCR.

### Statistical analysis

SPSS22.0 was adopted for all statistical analyses. Differences in characteristics between HCC patients and healthy controls were assessed using the Student's *t*-test or Pearson's χ^2^ test, and the Student's *t*-test was also carried out for difference analysis of between the two groups of experiments *in vivo* and *in vitro*. Pearson’s correlation analysis was performed to assess correlations between *SENP3-EIF4A1* and miR-9-5p/*ZFP36* in HCC samples. The ROC curve reflected the AUC values for exosomal *SENP3-EIF4A1* in plasma. *P*<0.05 indicated a statistically significant difference.

## Supplementary Material

Supplementary Figures
