# Supplementary material for: Exosome-transmitted long non-coding RNA SENP3-EIF4A1 suppresses the progression of hepatocellular carcinoma
Source: Aging (Albany NY). 2020 Jun 27;12(12):11550–67. doi: 10.18632/aging.103302 (PMC7343467; doi:10.18632/aging.103302)
Supplement: Supplementary Figures [file aging-12-103302-s001..pdf]

SUPPLEMENTARY FIGURES

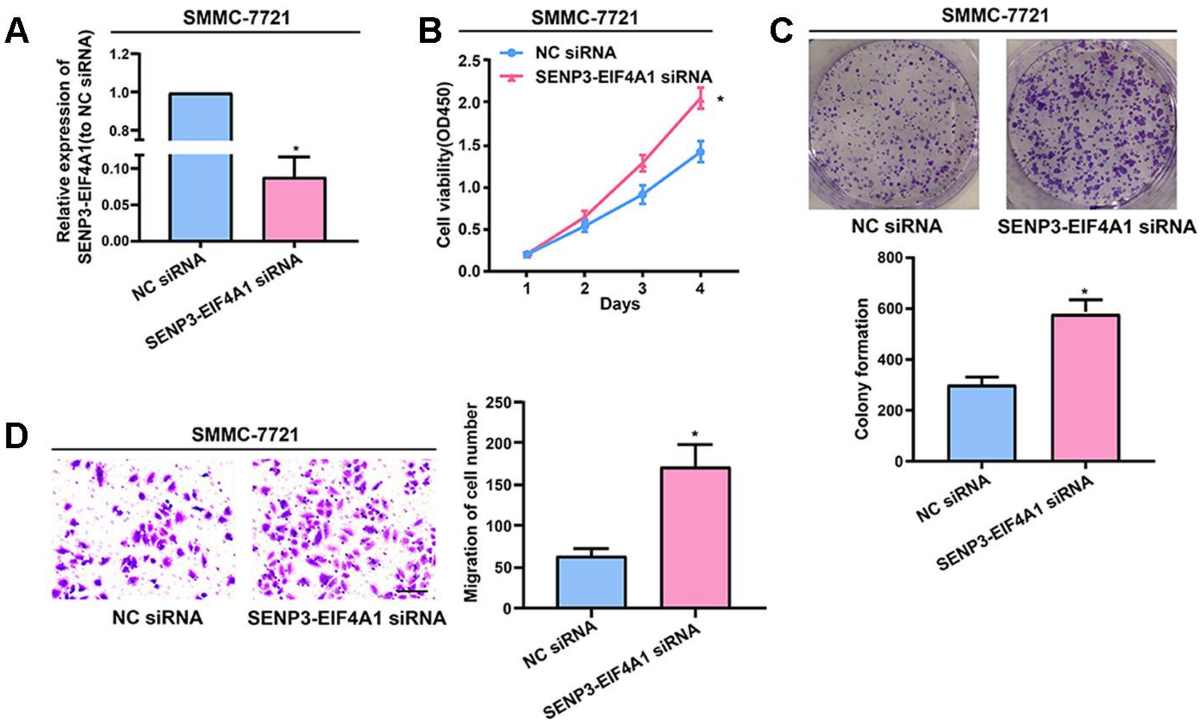

Supplementary Figure 1. Effect of *SENP3-EIF4A1* on HCC cellular phenotype. SMMC-7721 cells are transfected with *SENP3-EIF4A1* siRNA or negative control. (**A**) Detection of the mRNA level of *SENP3-EIF4A1* via qRT-PCR. (**B**) Examination of cell proliferation via CCK8 assays. (**C**) Determination of cell colony formation ability via a colony-forming growth assay. The colonies are counted and captured. (**D**) Representative images of migration assays of SMMC-7721 cells. The cells are counted. Results are shown as mean ± SD. *P<0.05. All of the experiments were performed in triplicate.

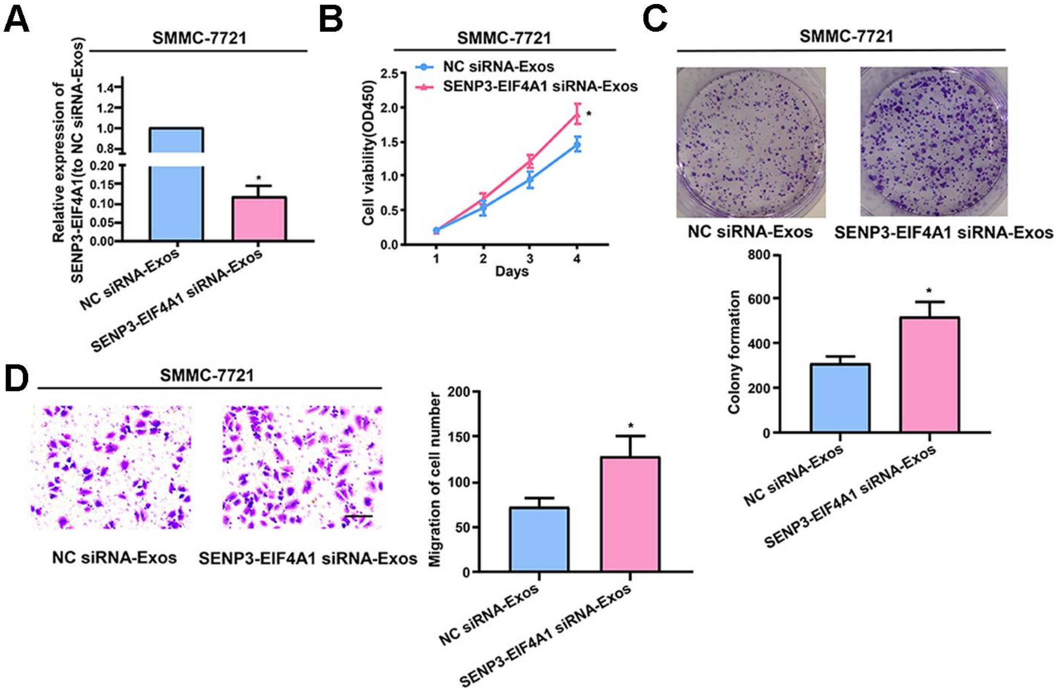

Supplementary Figure 2. Effect of exosomal *SENP3-EIF4A1* on HCC cellular phenotype. Exosomes separated from HL-7702 cells transfected with *SENP3-EIF4A1* siRNA or NC siRNA are named *SENP3-EIF4A1* siRNA-Exos or NC siRNA-Exos, respectively. After extraction, their exosomes are added to the SMMC-7721 cells for 24h. (**A**) Detection of the mRNA level of *SENP3-EIF4A1* via qRT-PCR. (**B**) Detection of cell proliferation by a CCK8 assay. (**C**) Examination of cell colony formation ability by a colony-forming growth assay. The colonies are counted and captured. (**D**) Representative images of migration assays of SMMC-7721 cells. The cells are counted. Results are shown as mean ± SD. **P*<0.05. All of the experiments were performed in triplicate.
